# Low-precision feature selection on microarray data: an information theoretic approach

**DOI:** 10.1007/s11517-022-02508-0

**Published:** 2022-03-22

**Authors:** Laura Morán-Fernández, Verónica Bolón-Canedo, Amparo Alonso-Betanzos

**Affiliations:** CITIC, Universidade da Coruña, A Coruña, Spain

**Keywords:** Microarray data, Low precision, Feature selection, Mutual information, Classification, Edge computing, Internet of Things

## Abstract

The number of interconnected devices, such as personal wearables, cars, and smart-homes, surrounding us every day has recently increased. The Internet of Things devices monitor many processes, and have the capacity of using machine learning models for pattern recognition, and even making decisions, with the added advantage of diminishing network congestion by allowing computations near to the data sources. The main restriction is the low computation capacity of these devices. Thus, machine learning algorithms capable of maintaining accuracy while using mechanisms that exploit certain characteristics, such as low-precision versions, are needed. In this paper, low-precision mutual information-based feature selection algorithms are employed over DNA microarray datasets, showing that 16-bit and some times even 8-bit representations of these algorithms can be used without significant variations in the final classification results achieved.

**Graphical Abstract:**
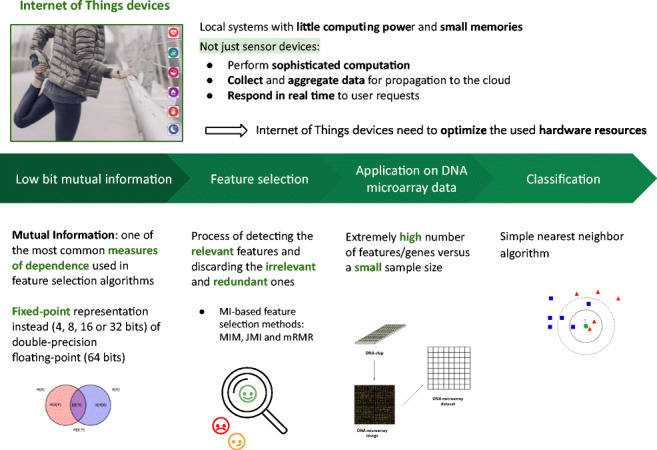
Graphical abstract

Graphical abstract

## Introduction

The need for efficient algorithms has been one of the goals in Computer Science. But during the last years we have assisted also to the growing tendencies in sensoring and monitoring of activities and processes, and thus, among others, to what are called Big Data, on the one hand, and Internet of Things (IoT), in the other. These two tendencies have given birth to research areas on Cloud Computing or Edge Computing. Due to the increasing communication costs of sending/receiving data from and to the cloud, there is lately a growing interest in performing ever more complex machine learning tasks on mobile and embedded devices, frequently in real-time. Thus, the objective is to optimize the use of hardware resources and power consumption while keeping algorithms’ accuracy comparable to the classical computations that use double-precision floating-point arithmetic.

Among the different machine learning methods, feature selection (FS) is a fundamental task, as it can help in reducing dimension and thus contributes for more understandable models. FS is a dimensionality reduction method that works by removing those features that are redundant and/or irrelevant and only keeping the relevant features (or genes, in this case). The main benefits of feature selection are reducing experimental costs, enhancing interpretability, speeding up computation, reducing memory and even improving model generalization.

However, feature selection is also a challenging task from the point of view of resource consumption, since a dataset with *m* features will produce 2^*m*^ − 1 candidate subsets. The vast majority of algorithms rely on searching over the feature space which is exhaustive, expensive and time-consuming. Meanwhile, due to the explosive growth of wireless communication technology and to the progressive reduction in the cost of electronic components, the number IoT devices has increased dramatically in recent years, as said above. In contrast to up-to-date computers, IoT devices need to optimize the use of hardware resources, so a possible solution is to adapt machine learning methods to work on low-precision (i.e., less than 64 bits).

On the other hand, and regarding application fields, during the last few decades, the emergence of microarray datasets has stimulated a new line of research, both in bioinformatics and in machine learning. This type of datasets poses an interesting challenge because of two reasons: (i) they have very small samples—often less than 100 patients—in contrast to a very high dimensionality—the number of features ranges in the order of thousands; and (ii) it has been shown that most features are not necessary to an accurate classification [12], so it is paramount to discover the relevant features to gather an understanding of the process. Thus, FS has become a must-do in dealing with these datasets [6].

In a previous work, we have proposed a low-precision mutual information feature selection procedure [27]. Mutual Information (MI) comes from the field of Information Theory and it is widely used in both machine learning and statistics. As a matter of fact, it is part of the popular method mininum Redundancy Maximum Relevance (mRMR), which is known to work very well with microarray data [30]. To the best of our knowledge, ours is the first and only attempt to adapt feature selection to low-precision, despite the expected benefits that it could add to embedded systems for on-device analysis.

The goal of the work described inhere is to apply low-precision mutual information feature selection on a challenging scenario: microarray data. Three different implementations will be tested (mutual information maximization, mRMR and joint mutual information), to check if the use of low-precision parameters is possible in datasets with such high dimensionality as microarrays.

The rest of the paper is organized as follows: Section 2 describes the state of the art of low-precision feature selection. Section 3 presents our low-precision mutual information approach. Section 4 describes the materials and methods used in the experiments, whose results are shown and analyzed in Section 5. Finally, Section 6 contains our concluding remarks and proposals for future research.

## State of the art

With the growing amount of information being generated at the edge, the demand for machine learning models that can be deployed on edge devices has also increased. Although most of the effort has been put on adapting deep learning models to work on edge devices, there are some works that have developed techniques for distributed training or compression and pruning of other machine learning methods. Wang et al. [36] presented a technique to train machine learning methods at the edge that uses gradient-based approaches (e.g., SVMs, K-means, linear regression or CNNs). ProtoNN is an algorithm designed by Gupta et al. [13] based on kNN that projects data to a lower dimensional space using a sparse-projection matrix in order to reduce storage requirements. ProtoNN has shown to be only 1–2% less accurate while consuming 1–2 orders of magnitude less memory. Also based on reducing the model size is Bonsai [19], a tree-based algorithm that significantly outperforms state-of-the-art techniques in terms of model size, accuracy, speed, and energy consumption. Finally, the researchers in [22] investigated the effects of parameter quantization and of reduced working precision on the accuracy of floating-point SVM classification.

As mentioned above, much effort has been made to adapt deep learning algorithms for training or inference on the edge, as depicted in several review works [28, 40]. One challenging option is to actually train the deep learning algorithms on the edge, for which federated learning is the most used approach [38]. Other works are focused on just deploying on the edge already trained models, so typical strategies are to reduce the number of trainable parameters and minimize the number of computations [17], or to reduce the size of the models by performing quantization^1^ or model compression^2^ [9, 10].

Since edge-devices have limited computing power, energy consumption is a critical factor, so recent research trends show that much effort is being put into compressing neural networks. Several papers have attempted this approach through quantization, which is able to lower the memory footprint and potentially speed up the computations. In relation to inference accuracy, many studies have shown that it is possible to achieve the same results with reduced precision of weights and activations [14, 24]. Regarding learning, Hubara et al. [18] introduced a method to train Quantized Neural Networks using extremely low precision and runtime activations, reaching an accuracy comparable to networks trained using 32 bits. The research of Yu et al. [39] presents a method of quantification with mixed data structure and proposes a hardware accelerator. This allows them to reduce the number of bits needed to represent neural networks from 32 to 5, also without affecting their accuracy. Banner et al. [3] introduced a 4-bit post training quantization approach with just a few percent accuracy degradation. Finally, the work of Sun et al. [33] shows that it is possible to train deep neural networks using only 4 bits with non-significant loss in accuracy while enabling significant hardware acceleration.

With regard to reducing energy consumption in feature selection, we can only find our own work in which we presented a limited bit depth mutual information that can be applicable to any feature selection method that uses internally the mutual information measure [25, 27], which will be detailed in the following section.

## Low-precision mutual information

### Background

Mutual Information (MI) comes from the field of Information Theory and it is widely used in both machine learning and statistics. One of its main uses is feature selection methods, and in fully supervised data, the features *X* are ranked using this measure, and the ones finally selected are those having the highest mutual information with the class label *Y*. The mutual information is defined as the expected logarithm of a ratio:

1 $$  I(X;Y) = \underset{x \in \mathcal{X}}{\sum} \underset{y \in \mathcal{Y}}{\sum} p(x,y) \ln \frac{p(x,y)}{p(x)p(y)} $$

where *p*(*x*,*y*) = *Pr*{*X* = *x*,*Y* = *y*} is the probability mass function of the joint distribution when the random variable *X* takes on the value *x* from its alphabet $\mathcal {X}$ and *Y* takes on $y \in \mathcal {Y}$, while *p*(*x*) = *Pr*{*X* = *x*} and *p*(*y*) = *Pr*{*Y* = *y*} are the probability mass functions of the marginal distributions. In this work, the function is calculated in natural logarithm, so returned units are “nats”. In practice we have to estimate this from data. This can be done by using the sample (maximum likelihood) estimates of the probabilities $\hat {p}$ and plug them in Eq. 1. This maximum likelihood estimator for the mutual information is consistent [29], and as a result we have:

2 $$ I(X;Y) \approx \hat{I}(X;Y) = \sum\limits_{x \in \mathcal{X}} \sum\limits_{y \in \mathcal{Y}} \hat{p}(x,y) \ln \frac{\hat{p}(x,y)}{\hat{p}(x)\hat{p}(y)} $$

In order to calculate this we need the estimated distributions $\hat {p}(x,y), \hat {p}(x),$ and $\hat {p}(y)$. The probability of any particular event *p*(*X* = *x*) is estimated by maximum likelihood, the frequency of occurrence of an event *X* = *x* divided by the total number of events.

**An illustrative example.** Let us consider a vector *Y* with 961 observations, in which the number of occurrences of an event *Y* = *y* is 4. The probability $\hat {p}(y)$ will be $\hat {p}(y)=4/961=0.004162330905307$, which is approximately zero. For real applications, it is not necessary to store all the decimal digits, which makes mutual information an interesting measure to explore low precision. Besides, as the Internet of Things devices market matures, we will likely see a movement away from double-precision floating-point (i.e., 64-bit representation) to limited approaches using a lower number of bits.

### Our approach

In information theoretic feature selection, the main challenge is to estimate the mutual information, for which it is necessary to estimate the probability distributions. Internally, it counts the occurrences of values within a particular group (i.e., its frequency). Based on Tschiatschek et al.’s [34] work for approximately computing probabilities, we investigated mutual information with limited number of bits by considering this measure with low-precision counters in a previous work [27]. Instead of the 64-bit resolution used typically by the standard hardware platforms, a fixed-point representation was targeted with *bi* as the number of integer bits and *bf* as the number of fractional bits. The motivation to move to fixed-point arithmetic is twofold: (i) these bit representation compute units are typically faster and consume far less hardware resources and power than the conventional floating-point computations and (ii) low-precision data representation reduces the memory footprint, enabling larger models to fit within the given memory capacity and lowering the bandwidth requirements.

Besides, since mutual information parameters are typically represented in the logarithmic domain, we compute the number of occurrences of an event and use a lookup table to determine the logarithm of the probability of a particular event. The lookup table is indexed in terms of number of occurrences of an event (individual counters) and the total number of events (total counter) and stores values for the logarithms in the desired low-precision representation. To limit the maximum size of the lookup table and the bit-width required for the counters, we assumed some maximum integer number *M*. The lookup table *L* is pre-computed such that:

3 $$  L(i,j)= \left[\frac{ln(i/j)}{q} \right]_{R} \cdot q $$

where [⋅]_*R*_ denotes rounding to the closest integer, *q* is the quantization interval of the desired fixed-point representation (2^−*bf*^), *ln*(⋅) denotes the natural logarithm, and where the counters *i* and *j* are in the range {0,...,*M* − 1}.

Given certain specific data, the individual counters ${c_{j}^{i}}$ and the population *C* are computed according to Algorithm 1. Following the fixed-point representation, we assumed some maximum integer number *M*, where *M* = 2^(*bf*+*bi*)^ − 1. After calculating the cumulative count *C*, we ensure that it is in range. Also, we divide by two the individual counters *c*_*i*_ when *C* reaches its maximum value. 
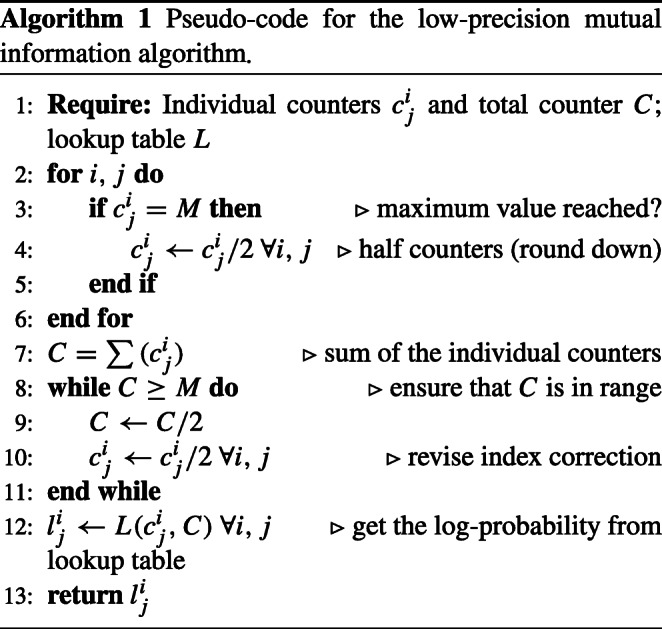


## Materials and methods

### DNA microarray datasets

Microarray technology is used to collect information from tissue and cell samples regarding gene expression differences that could be useful for diagnosing diseases. During the last two decades, the advent of this type of datasets has stimulated a new line of research both in bioinformatics and in machine learning. Although there are usually very small samples (often less than 100 patients) for training and testing, the number of features in the raw data ranges from 2000 to 25,000. A typical classification task is to separate healthy patients from cancer patients based on their gene expression profile (binary approach). There are also datasets in which the goal is to distinguish among different types of tumours (multiclass aproach), making the task even more complicated. Therefore, microarray data poses a serious challenge for machine learning researchers. Having so many features relative to so few samples creates a high likelihood of finding false positives due to chance (both in finding relevant genes and in building predictive models). Thus, it becomes necessary to find robust methods to validate the models and assess their likelihood.

Besides, several studies have shown that most genes measured in a DNA microarray experiment are not relevant in the accurate classification of different classes of the problem [12]. To avoid the problem of the *curse of dimensionality*, feature selection plays a crucial role in DNA microarray analysis, so that the learning algorithm focuses only on those aspects of the training data useful for analysis and future prediction. Apart from the mismatch between dimensionality and sample size, microarray data have other particularities such as the imbalance of the data, their complexity, the presence of overlapping, or the so-called dataset shift [6]. Table 1 profiles the main characteristics of the 17 DNA microarray datasets used in this research in terms of the number of samples, features and classes [2, 7, 26, 32].

**Table 1:** Characteristics of the 17 DNA microarray datasets. It shows the number of samples (#sam.), features (#feat.) and classes (#cl.)

Dataset	#sam.	#feat.	#cl.	Dataset	#sam.	#feat.	#cl.
9-tumors	60	5726	9	Gli85	85	22283	2
11-tumors	174	12533	11	Leukemia-1	72	5327	3
Brain	21	12625	2	Leukemia-2	72	11225	3
Brain-tumor-1	90	5920	5	Lung-cancer	203	12600	5
Brain-tumor-2	50	10367	4	Ovarian	253	15154	2
CLL-SUB-111	111	11340	3	Smk	187	19993	2
CNS	60	7129	2	SRBCT	83	2308	4
Colon	62	2000	2	TOX-171	171	5748	4
DLBCL	47	4026	2				

### MI-based feature selection methods

Mutual information definition is useful within the context of feature selection because it gives a way to quantify the output vector. Thus, there exist in the literature several feature selection methods based on mutual information measures. Most methods define heuristic functionals to assess feature subsets combining definitions of relevant and redundant features. Among the different information theoretic methods, we have chosen three to evaluate our low-precision mutual information approach, each of them making different assumptions. For example, Mutual Information Maximization quantifies only the relevancy, minimum Redundancy Maximum Relevance the relevancy and redundancy, while the Joint Mutual Information the relevancy, the redundancy and the complementarity [8].

- **Mutual Information Maximization** (MIM) [23] ranks the features by their mutual information score, and selects the top *k* features, where *k* is decided by some predefined need for a certain number of features or some other stopping criterion. An important limitation is that this assumes that each feature is independent of all other features and effectively ranks the features in descending order of their mutual information content. Thus, this approach does not take into account the redundancy between the features.
- **minimum Redundancy Maximum Relevance** (mRMR) [30] feature selection method selects features that have the highest relevance with the target class and are also minimally redundant, i.e., it selects features that are maximally dissimilar to each other. Both optimization criteria (maximum-relevance and minimum-redundancy) are based on mutual information.
- **Joint Mutual Information** (JMI) [37] is another feature selection method based on mutual information, and it adopts a new criterion to evaluate the candidate features. JMI chooses the feature that has the maximum cumulative summation of joint mutual information with the selected features in each step and adds it to the subset *S* until the number of selected features reaches *k*.

Let us assume that we have a dataset of *m* samples and *n* features and that we wish to select the top-*k*. Table 2 shows the theoretical complexity of the three methods described above [31].

**Table 2:** Theoretical complexity of the three feature selection methods focus of this work

Method	Complexity
MIM	$\mathcal {O}(k \cdot m \cdot n)$
mRMR	$\mathcal {O}(k^{2} \cdot m \cdot n)$
JMI	$\mathcal {O}(k^{2} \cdot m \cdot n)$

## Results

In this section we empirically evaluate our low-precision mutual information method described in Section 3. Among the different methods that use internally the mutual information measure, we have chosen feature selection since this process has a key role to play in helping to identify the specific genes that enhance classification accuracy in DNA microarray data. As said above, there is a large number of feature selection methods that use mutual information as a metric to establish the importance of the features, thus their performance depending on the accuracy obtained by the mutual information step. In this work, we have implemented our limited bit depth mutual information in the MIM, mRMR and JMI filters methods due to their popularity and good results in the machine learning area. In order to estimate mutual information of continuous features, the DNA microarray datasets were discretized, using an equal-width strategy into 10 bins. After the feature selection process the original (undiscretized) datasets were used to classify the test data.

In the following sections, we investigate the questions: “how similar are the rankings obtained by the different low-precision MI-based feature selection approaches?” and “which is the impact of these rankings on classification?”. To address these questions, we use the 17 DNA microarray datasets detailed in Table 1. Experiments were executed in the Matlab2020a and Weka [15] environments, using default values for the parameters.

### How similar are the rankings obtained by the different low-precision MI-based feature selection approaches?

In this subsection, we will evaluate the similarity between the feature rankings obtained by the 64-bit mutual information and the low-precision versions (using fixed point representations with 4, 8, 16 and 32 bits) after performing the MIM, mRMR and JMI feature selection methods. To address this study, we show the true positive rate (TPR), which measures the proportion of features that are correctly identified as such, using the full mutual information version (64 bits) as the ideal ranking. In high dimensional datasets, like DNA microarray data, it is common to focus only on the top features, so in these experiments we compared only the *k* top features, with *k* = 5,10,20,30,40 and 50.

As can be seen from the experimental results illustrated in Table 3, the lowest values of the low-precision approach using 4 bits show that the correlation between its selected features and the ideal ranking is quite poor in the three information theoretic methods. However, from 8 bits on, all the approaches achieved a TPR close to 1, which means that the features selected by these low-precision approaches are very similar to those selected by the full version using 64 bits. It can also be observed that, in general, by increasing the number of selected features, the TPR is higher.

**Table 3:** Average True Positive Rate of the low-precision approaches using the three different MI-based feature selection methods over the 17 microarrays datasets

	#bits	#features					
		5	10	20	30	40	50
MIM	4	0.059	0.094	0.106	0.096	0.096	0.099
	8	0.906	0.918	0.915	0.912	0.919	0.905
	16	0.976	1.000	0.988	0.990	0.987	0.993
	32	1.000	1.000	0.997	1.000	0.993	0.996
mRMR	4	0.024	0.047	0.065	0.084	0.097	0.099
	8	0.765	0.788	0.815	0.871	0.885	0.905
	16	0.953	0.976	0.988	0.994	0.993	0.993
	32	0.976	0.994	0.994	0.996	0.997	0.996
JMI	4	0.024	0.035	0.029	0.045	0.046	0.044
	8	0.729	0.741	0.838	0.896	0.916	0.924
	16	0.882	0.918	0.909	0.945	0.962	0.972
	32	0.859	0.871	0.924	0.953	0.968	0.972

**Fig. 1:**
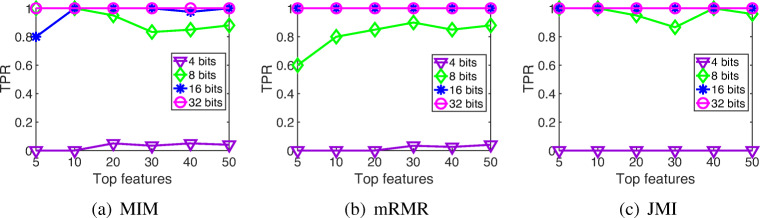
True positive rate of the different low-precision approaches on Colon dataset. **a** MIM. **b** mRMR. **c** JMI

**Fig. 2:**
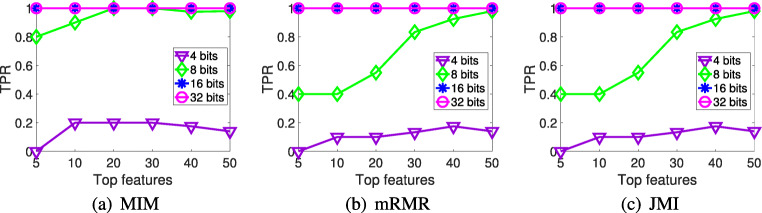
True positive rate of the different low-precision approaches on Ovarian dataset. **a** MIM. **b** mRMR. **c** JMI

**Fig. 3:**
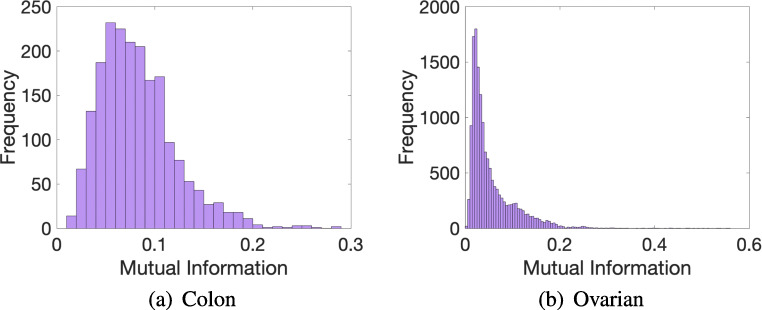
Histogram of frequency distribution values of mutual information of Colon and Ovarian microarrays. Note that the axes are scaled differently for each dataset. **a** Colon. **b** Ovarian

Trying to understand the possible effect that the size of the datasets could have on our results, we analyzed the TPR in two different DNA microarrays: Colon (62 samples and 2000 features) and Ovarian (253 samples and 15,154 features). As can be seen in Figs. 1 and 2, as the number of samples and features of the dataset increases, the performance of our low-precision version using 8 bits decreases. Regarding the 4-bit low-precision version, it achieved higher values of TPR in Ovarian dataset. This could be happening because, despite the fact that the Ovarian dataset clearly has a greater number of features, it also presents higher values of mutual information than in thev case of the Colon dataset (Fig. 3). Remember that, in terms of maximum relevance, the selected features are individually required to have the largest mutual information with the class label, reflecting the largest dependency on the target class.

Finally, we compared the results between the different feature selection methods. It is worth noticing that the univariate filter MIM, which takes into account only the individual relevance of each feature, performs better than the multivariate filters mRMR and JMI, which take into account feature dependencies. The information loss when reducing the number of bits affects the results much more than in the case of the less complex univariate methods. Besides, it can be seen that JMI performs better—in some cases—than MIM and mRMR when 8 bits are used. This could be because JMI criterion has the best trade-off in terms of stability and flexibility over other feature selection methods based on Information Theory due to its nature (it balances the relevancy and redundancy terms and includes the conditional redundancy) [8].

### Which is the impact of these rankings on classification?

Once feature selection has been carried out, and in order to estimate whether the low-precision mutual information in the MIM, mRMR and JMI methods might affect classification, a study using two classifiers belonging to different families was performed. At this point, it is necessary to clarify that including classifiers in our experiments is likely to obscure the experimental observations related to feature selection performance using a limited number of bits, since they have their own assumptions and particularities. It has been shown that certain classifiers can obtain outstanding accuracy levels even when the feature ranking is not optimal [5]. Therefore, in these experiments, we used a simple nearest neighbor algorithm (with number of neighbors *k* = 3) [1], since it makes few assumptions about the data and we avoid the need for parameter tuning, and a linear support vector machine (SVM) [35], due to its superiority in performance over other classifiers in this specific domain of microarray datasets [6, 16], as well as a boosting algorithm (LogitBoost) [11] . To estimate the error rate we computed 3 × 5-fold cross-validation (i.e., 3 repetitions of a cross-validation with 5 folds), including both feature selection and classification steps in a single cross-validation loop [21].

Tables 4, 5 and 6 show the average classification accuracy (between 0 and 100%) obtained by 3-NN, SVM and LogitBoost classifiers when using the feature ranking built with the 4, 8, 16, 32 and 64 bit versions by the MIM, mRMR and JMI feature selection methods, respectively. As can be seen for the three different information theoretic methods, the 8, 16 and 32 low-precision versions achieved very competitive results—in some cases even better—than the baseline 64-bit approach. Besides, we can see that the classification accuracy improves as the number of features increases. Remember that, in the case that the top 50 features are selected, the number of features used to train the model will be not even 3% of the number of features in the original microarray dataset.

**Table 4:** Average classification accuracy (*%*) over the 17 microarray datasets for MIM method

	#bits	#features					
		5	10	20	30	40	50
3NN	4	62.377	65.459	67.210	69.693	70.979	71.695
	8	70.983	73.518	**75.288**	75.443	**76.732**	76.737
	16	70.963	73.315	74.944	**75.840**	76.552	**77.219**
	32	**71.133**	73.524	74.879	75.785	76.168	76.851
	64	70.800	**73.681**	75.076	75.644	76.070	76.753
SVM	4	59.188	62.753	68.687	71.996	74.691	75.057
	8	68.857	**73.545**	**76.667**	77.995	79.067	79.817
	16	69.097	73.335	76.465	**78.309**	**79.613**	79.876
	32	**69.103**	73.396	76.502	77.745	79.531	**80.043**
	64	69.064	73.396	76.600	78.143	79.178	**80.043**
LogitBoost	4	62.973	66.764	70.303	71.025	72.627	73.822
	8	67.816	**71.436**	72.662	**74.376**	74.688	**75.927**
	16	**67.901**	71.410	73.248	73.744	**75.431**	75.566
	32	67.858	71.431	**73.328**	73.776	75.377	75.624
	64	**67.901**	71.392	73.241	73.950	75.377	75.592

For each classifier and number of features, highest accuracy rates highlighted in bold

**Table 5:** Average classification accuracy (*%*) over the 17 microarray datasets for mRMR method

	#bits	#features					
		5	10	20	30	40	50
3NN	4	60.103	63.888	67.487	69.237	70.630	71.695
	8	71.565	73.800	75.894	76.639	77.262	76.737
	16	**71.972**	73.686	76.099	**76.809**	**77.691**	**77.219**
	32	71.778	**73.822**	76.352	76.538	77.423	76.851
	64	71.430	73.583	**76.450**	76.538	77.344	76.753
SVM	4	57.553	61.765	68.818	71.354	73.661	74.996
	8	69.579	**74.881**	**77.566**	**78.856**	79.644	79.743
	16	69.815	74.068	77.428	78.311	79.390	79.922
	32	69.700	73.980	77.423	78.401	**79.818**	**80.051**
	64	**69.842**	74.034	77.325	78.205	**79.818**	**80.051**
LogitBoost	4	60.933	64.032	68.071	69.594	72.119	73.702
	8	**70.327**	72.551	74.041	**74.687**	75.104	**76.031**
	16	70.134	**72.734**	74.346	74.194	75.103	75.517
	32	69.846	72.719	74.684	74.031	**75.117**	75.676
	64	70.184	72.664	**74.727**	74.149	75.063	75.523

For each classifier and number of features, highest accuracy rates highlighted in bold

**Table 6:** Average classification accuracy (*%*) over the 17 microarray datasets for JMI method

	#bits	#features					
		5	10	20	30	40	50
3NN	4	60.463	64.579	67.060	68.889	70.330	70.632
	8	67.823	70.530	72.545	73.691	73.801	**74.147**
	16	68.060	**70.754**	**72.913**	74.262	74.131	73.751
	32	67.967	70.692	72.801	74.110	74.297	73.805
	64	**68.123**	70.354	72.755	**74.602**	**74.405**	73.703
SVM	4	55.784	60.257	64.841	68.408	70.856	72.044
	8	67.750	71.653	75.892	76.907	78.193	78.159
	16	67.756	71.598	75.587	76.460	**78.245**	**78.796**
	32	68.161	71.898	75.632	**76.934**	78.236	78.343
	64	**68.210**	**72.424**	76.079	77.621	78.081	78.542
LogitBoost	4	61.420	66.392	70.438	71.468	72.617	74.232
	8	**67.467**	69.415	72.782	72.683	73.498	74.050
	16	66.630	69.607	**72.950**	72.761	**73.620**	74.413
	32	66.945	69.430	71.653	**72.976**	73.520	**74.427**
	64	66.522	**69.969**	72.635	72.789	73.253	74.402

For each classifier and number of features, highest accuracy rates highlighted in bold

To explore the statistical significance of our classification results, and due to the drawbacks of the traditional tests of contrast of the null hypothesis pointed up by [4], we have chosen to apply the Bayesian hypothesis test [20]. In this type of analysis, a previous step is needed, which consists in the definition of the Region of practical equivalence (Rope). Two methods are considered practically equivalent in practice if their mean differences given a certain metric are less than a predefined threshold. In our case, we will consider two methods as equivalent if the difference in error is less than 1*%*. For the whole benchmark and each pair of methods, we calculated the probability of the three possibilities: (i) low-precision version wins over full version (64-bit) with a difference larger than *rope*, (ii) full version wins over low-precision with a difference larger than *rope*, and (iii) the difference between the results are within the *rope* area. If one of these probabilities is higher than 95*%*, we consider that there is a significant difference.

**Fig. 4:**
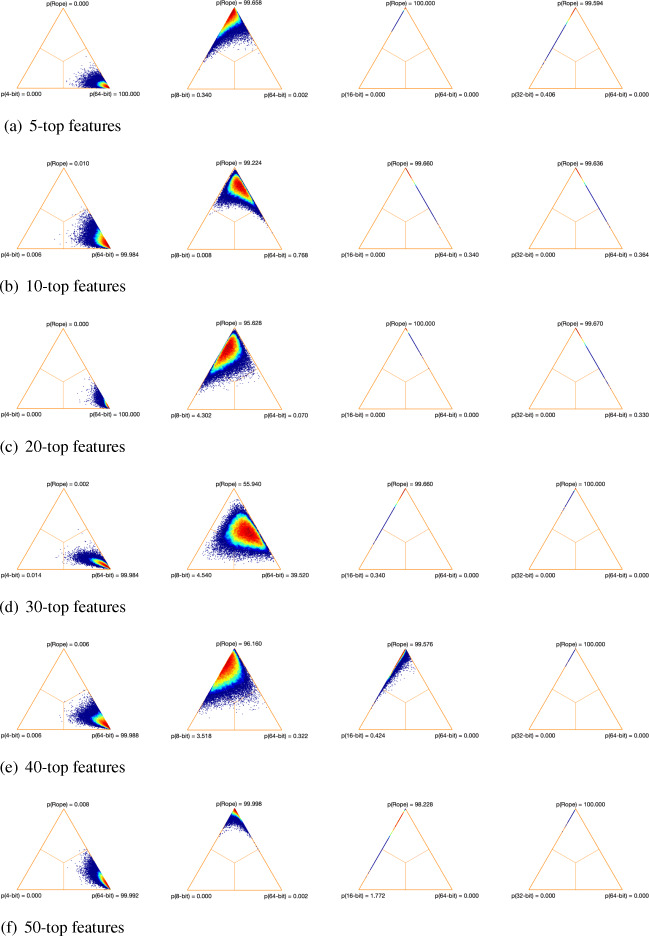
Simplex graphs for pair comparison of each low-precision version and the baseline full version over the 17 microarray datasets for MIM and 3NN classifier using Bayesian hierarchical tests: low-precision version (left) and full version (right). **a** 5-top features. **b** 10-top features. **c** 20-top features. **d** 30-top features. **e** 40-top features. **f** 50-top features

**Fig. 5:**
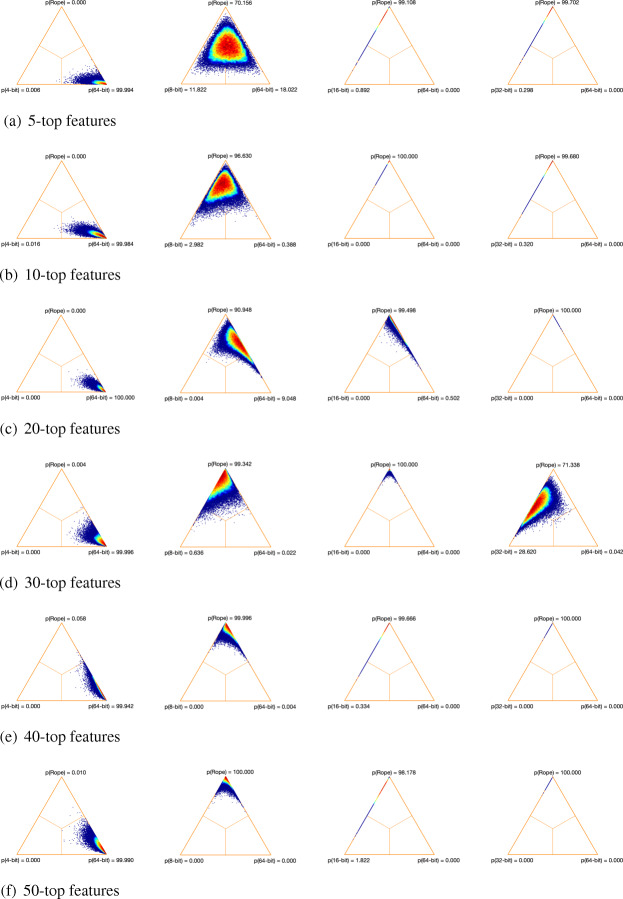
Simplex graphs for pair comparison of each low-precision version and the baseline full version over the 17 microarray datasets for mRMR and 3NN classifier using Bayesian hierarchical tests: low-precision version (left) and full version (right). **a** 5-top features. **b** 10-top features. **c** 20-top features. **d** 30-top features. **e** 40-top features. **f** 50-top features

**Fig. 6:**
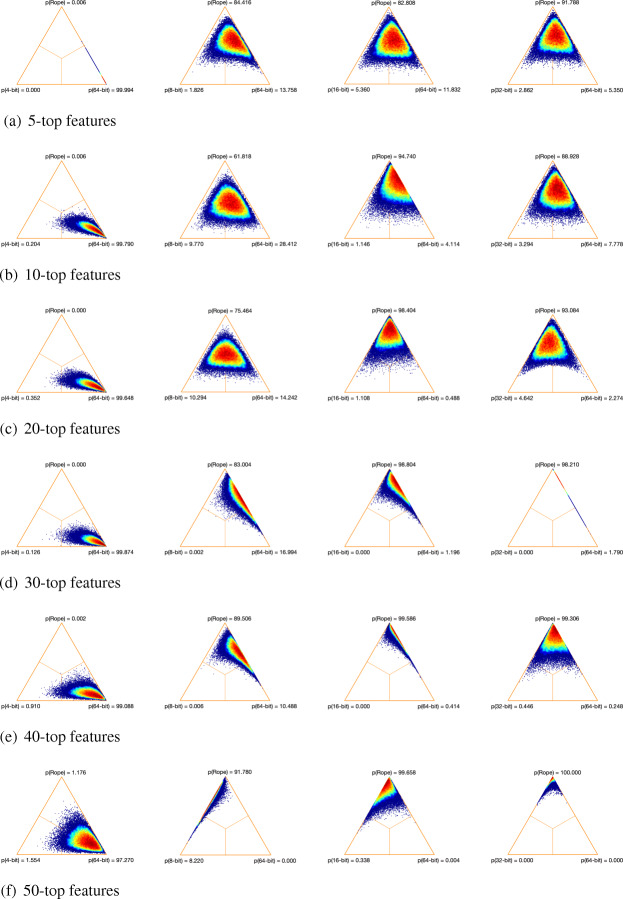
Simplex graphs for pair comparison of each low-precision version and the baseline full version over the 17 microarray datasets for JMI and 3NN classifier using Bayesian hierarchical tests: low-precision version (left) and full version (right). **a** 5-top features. **b** 10-top features. **c** 20-top features. **d** 30-top features. **e** 40-top features. **f** 50-top features

Figures 4, 5 and 6 show the distribution of the differences between each pair of methods using simplex graphs. Since analyzing specific aspects related to classification is not the goal of this paper, we only show the results for the 3-NN classifier (because it makes less assumptions about the data than SVM and LogitBoost). As can be seen, regardless of the feature selection method, the low-precision versions with 8, 16 and 32 bits are practically equivalent to the 64-bit baseline version (the highest probability values are obtained by *rope*). In the case of the 4-bit version, and as we have been observing in the results obtained so far, here there is statistical significance with respect to the 64-bit version, since the probability that the full approach using 64 bits wins over the 4-bit—represented in the figures as p(64-bit)—is greater than 95% in all the cases.

Finally, Table 7 shows the runtime required by the three classification algorithms. In terms of classification accuracy, the best results were obtained by the SVM classifier. However, in the case of comparing them by their computational time, a good choice would be the 3NN classifier. This model has a slightly lower accuracy than the other two classifiers, but requires less than 1/2 of the time to classify. In addition, it can be observed how the computation time increases in the microarray datasets with the largest number of samples and classes (i.e., 9-tumors, 11-tumors, Brain-tumor-1 and Lung-cancer).

**Table 7:** Runtime (s) for the classification algorithms tested

Dataset	3NN	SVM	LogitBoost	Dataset	3NN	SVM	LogitBoost
9-tumors	0.303	1.080	0.741	Gli85	0.312	0.327	0.627
11-tumors	0.321	1.405	0.851	Leukemia-1	0.309	0.481	0.629
Brain	0.286	0.310	0.628	Leukemia-2	0.310	0.481	0.629
Brain-tumor-1	0.310	0.704	0.633	Lung-cancer	0.338	0.704	0.633
Brain-tumor-2	0.304	0.598	0.630	Ovarian	0.338	0.361	0.627
CLL-SUB-111	0.319	0.482	0.628	Smk	0.333	0.353	0.628
CNS	0.304	0.317	0.627	SRBCT	0.321	0.604	0.631
Colon	0.302	0.323	0.627	TOX-171	0.338	0.607	0.632
DLBCL	0.298	0.311	0.627				

Runtime is calculated as the average of the 3 repetitions of a cross-validation with 5 folds

To sum up, these experimental results show that, with a small number of bits (32, 16 and even 8) the rankings change, but this variation does not affect significantly the classification performance, since this measure is the ultimate form of evaluation of the goodness of a ranking feature selection method. However, this method has also some drawbacks. If there is a short distance between the population values of the mutual information, our low-precision approach will not be adequate. Besides, we will require additional bits as the number of features/samples of the dataset grows. Nevertheless, it is worth noting that our low-precision technique was created to evaluate data at the user level. In the case of dealing with large data, most likely these will be acquired from a variety of sources, and it will be processed either by more powerful central processors or disseminated over multiple nodes for further analysis.

## Conclusions

Driven by the proliferation of mobile computing and Internet of Things, in this work we have applied mutual information using low-precision parameters within a feature selection procedure. The obtained results over 17 microarray datasets demonstrated that 8-bit representations were sufficient to obtain feature rankings similar to those of double floating-point precision parameters and thus opening the door for the use of feature selection in Internet of Things devices that minimize the energy consumption and carbon emissions. Regarding the three feature selection methods used to test our low-precision mutual information, we have found that MIM was the most appropriate for this challenging scenario, taking into account not only its performance in classification but also its computational complexity.

As future research, we plan to develop other feature selection methods in low-precision, such as those based on distances (ReliefF) or on correlations. It would be also interesting to apply other strategies to represent data with a low number of bits, such as dynamic fixed point, and different techniques for rounding.

## Footnotes

1 Technique that reduces arithmetic complexity by decreasing the number of bits required to represent each weight.

2 Technique that reduces the number of model parameters and therefore improves storage and computing time.
